# Therapeutic Implications of Tumor Microenvironment in Lung Cancer: Focus on Immune Checkpoint Blockade

**DOI:** 10.3389/fimmu.2021.799455

**Published:** 2022-01-07

**Authors:** Carlo Genova, Chiara Dellepiane, Paolo Carrega, Sara Sommariva, Guido Ferlazzo, Paolo Pronzato, Rosaria Gangemi, Gilberto Filaci, Simona Coco, Michela Croce

**Affiliations:** ^1^ UO Clinica di Oncologia Medica, IRCCS Ospedale Policlinico San Martino, Genova, Italy; ^2^ Dipartimento di Medicina Interna e Specialità Mediche (DIMI), Università degli Studi di Genova, Genova, Italy; ^3^ Lung Cancer Unit, IRCCS Ospedale Policlinico San Martino, Genova, Italy; ^4^ Dipartimento di Patologia Umana, University of Messina, Messina, Italy; ^5^ SuPerconducting and Other INnovative Materials and Devices Institute, Consiglio Nazionale delle Ricerche (CNR-SPIN), Genova, Italy; ^6^ Life Science Computational Laboratory (LISCOMP), IRCCS Ospedale Policlinico San Martino, Genova, Italy; ^7^ UO Oncologia Medica 2, IRCCS Ospedale Policlinico San Martino, Genova, Italy; ^8^ UO Bioterapie, IRCCS Ospedale Policlinico San Martino, Genova, Italy

**Keywords:** NSCLC, PD-1/PD-L1, CTLA-4, tumor microenvironment (TME), immune checkpoint inhibitors, dysfunctional T cells, immunotherapy

## Abstract

In the last decade, the treatment of non-small cell lung cancer (NSCLC) has been revolutionized by the introduction of immune checkpoint inhibitors (ICI) directed against programmed death protein 1 (PD-1) and its ligand (PD-L1), or cytotoxic T lymphocyte antigen 4 (CTLA-4). In spite of these improvements, some patients do not achieve any benefit from ICI, and inevitably develop resistance to therapy over time. Tumor microenvironment (TME) might influence response to immunotherapy due to its prominent role in the multiple interactions between neoplastic cells and the immune system. Studies investigating lung cancer from the perspective of TME pointed out a complex scenario where tumor angiogenesis, soluble factors, immune suppressive/regulatory elements and cells composing TME itself participate to tumor growth. In this review, we point out the current state of knowledge involving the relationship between tumor cells and the components of TME in NSCLC as well as their interactions with immunotherapy providing an update on novel predictors of benefit from currently employed ICI or new therapeutic targets of investigational agents. In first place, increasing evidence suggests that TME might represent a promising biomarker of sensitivity to ICI, based on the presence of immune-modulating cells, such as Treg, myeloid derived suppressor cells, and tumor associated macrophages, which are known to induce an immunosuppressive environment, poorly responsive to ICI. Consequently, multiple clinical studies have been designed to influence TME towards a pro-immunogenic state and subsequently improve the activity of ICI. Currently, the mostly employed approach relies on the association of “classic” ICI targeting PD-1/PD-L1 and novel agents directed on molecules, such as LAG-3 and TIM-3. To date, some trials have already shown promising results, while a multitude of prospective studies are ongoing, and their results might significantly influence the future approach to cancer immunotherapy.

## 1 Introduction

In the last decades, a remarkable shift in the clinical management of non-small cell lung cancer (NSCLC) patients has been driven by the introduction of immune checkpoint inhibitors (ICI) targeting the axis involving programmed death protein 1 (PD-1) and its ligand (PD-L1). The introduction of these agents brought to unprecedented durability in the responses compared to chemotherapy. Notably, the most relevant benefit with single-agent ICI in NSCLC is observed in the case of patients whose tumor is characterized by high expression of PD-L1 (≥50%). Indeed, the anti-PD-1 agents pembrolizumab and cemiplimab, as well as the anti-PD-L1 agent atezolizumab, have achieved improved outcomes in terms of response and survival compared to chemotherapy in randomized phase III trials involving previously untreated patients affected by advanced NSCLC with high PD-L1 expression; conversely when PD-L1 expression is lower than 50% the advantage of PD-1 or PD-L1 inhibitors employed as single agent in first-line over platinum-based chemotherapy is limited, and this observation was confirmed in sub-group analyses of patients with PD-L1 between 1-49% enrolled in the KEYNOTE 042 and IMPOWER 110 trials (1–4). In order to improve the outcomes of patients with low or absent PD-L1 expression, anti-PD-1/PD-L1 agents have been employed in combination with either chemotherapy or with other ICI, such as agents targeting the cytotoxic T-lymphocyte-associated protein 4 (CTLA-4). In the randomized, phase III KEYNOTE 189 and KEYNOTE 407 trials, which involved patients with advanced non-squamous and squamous NSCLC, respectively, the addition of pembrolizumab to platinum-based chemotherapy resulted in improved outcomes in terms of response and survival over chemotherapy alone (5, 6). Notably, the advantage deriving from the combination of immunotherapy and chemotherapy was independent from the expression of PD-L1, including those patients whose tumor did not express PD-L1 at all (7, 8). More recently, the combination of the anti-PD-1 agent nivolumab and anti-CTLA-4 agent ipilimumab associated with two cycles of platinum-based chemotherapy achieved improved outcomes compared to first-line chemotherapy in the randomized, phase III CheckMate 9LA trial. Even in this case, the experimental combination achieved superior results irrespective of PD-L1 expression (7).

In spite of these impressive results, patients receiving ICI, either alone or as part of combination regimens, are destined to eventually experience disease progression associated with acquired resistance; furthermore, a non-negligible proportion of patients receiving ICI do not respond to treatment in spite of high PD-L1 expression. Indeed, response rate with single-agent pembrolizumab was 44.8% in KEYNOTE 024 (hence more than half of the patient population did not achieve partial response) (2); furthermore, in EMPOWER-LUNG 1, 18% of the patients randomized in the cemiplimab arm experienced disease progression as best response during treatment in spite of high PD-L1 expression (1). Hence, new combination approaches are warranted. Tumor microenvironment (TME) represents an element of increasing interest for the development of cancer immunotherapy as potential source of predictive factors for treatment with ICI or even as an additional therapeutic target by itself. TME consists of a heterogeneous population of cancer cells, immune cells, vessels, stroma, signaling mediators and extracellular matrix proteins (8). The presence of a chronic inflammatory environment in lung cancer (9) may alter or deviate immune cell differentiation, resulting in an imbalance of anti-tumor activity, thus favoring tumor evasion (8) and later on, resistance to ICI (10). In this context, TME might represent a relevant source of predictive biomarkers for ICIs, as well as a potential target for novel therapeutic strategies. Therefore, in this review we will point out the role of TME in the treatment of NSCLC with immunotherapy, either as a predictor of benefit from currently employed ICI or as therapeutic target from investigational agents. Furthermore, we will explore the potential impact of combinations including “classic” ICI and novel agents under clinical investigation. To this aim, we evaluated indexed publications on PubMed and abstracts presented at the most relevant scientific meetings.

## 2 Tumor Microenvironment

Studies on NSCLC TME based on histological and immunological analyses of the primary tumor have been difficult due to the limited availability of tissue because the majority of patients are diagnosed in advanced disease and are therefore inoperable. Nevertheless, different studies described a TME characterized by the presence of tumor infiltrating lymphocytes (TILs), which have been exploited to define prediction tools for patient’s survival and response to therapy. The presence of lymphocytes in the tumor area represents an independent prognostic factor for patient’s survival, with intense lymphocytic infiltration predicting longer survival (11, 12). In particular, CD8+T cells and M1-macrophages correlate with positive prognosis (12). The distribution of lymphocytes within the tumor evaluated through tissue microarrays revealed that high density of T lymphocytes (CD4+ and CD8+) in the tumor stroma correlated with better prognosis (12, 13). Beside this, it has been suggested that the presence of high density CD8+T cells in resected NSCLC may be considered as an additional marker to the tumor–node–metastasis classification (TNM-Immunoscore) (14, 15).

It is getting clearer that the reasons for the resistance to ICI must be sought in the tumor tissue, in the complex network of interactions that exist between tumor cells and TME (10). The presence of TILs, macrophages and dendritic cells (DC) may recall a hot TME potentially responsive to immunotherapy. Unfortunately, only a proportion of patients possess a hot TME, while more frequently cold (very few TILs) or ‘altered’ (TILs mainly at the edge of the tumor) TME have been observed (16). Spatial histology combined with exome and RNA-sequencing analyses on 100 patients from the TRACERx cohort helped to define that tumors with more than one immune cold region had a higher risk of relapse, regardless of tumor size and stage (17). Low TILs are also correlated with limited efficacy of ICI treatment and resistance to immunotherapy (14).

### 2.1 T and NK Cells Exhaustion

NSCLC is characterized by high levels of somatic non-synonymous mutations defined as tumor mutation burden (TMB), with higher numbers of mutations in metastases than in primary lung tumors (18–20). Mutations may originate neo antigens, which may be recognized by cytotoxic T cells in the TME, resulting in the development of an antitumor response. Although high infiltrated tumors might be advantaged in recognizing neo antigens, the presence of high TILs rather immunosuppressive or dysfunctional abolishes the possibility of that responses. In a recent published paper CD8+PD-L1+ TILs were associated with increased tumor burden constituting a hot but immunosuppressive TME, but patients with these characteristics were more likely to obtain a good response to anti-PD-1 therapy (21, 22). Using single-cell transcriptomics, Caushi et al., studied the transcriptional programs of mutation-associated neoantigens (MANA)-specific TILs from tumors of 20 patients, which received nivolumab +/- ipilimumab, enrolled in the clinical trial NCT02259621. MANA-specific CD8+ T cells were more numerous in the tumor than in normal lung. MANA-specific T cells from responsive patients showed higher expression of genes associated with memory (*IL7R* and *TCF7*) and effector functions (*GZMK*), while MANA-specific T cells from non-responsive patients expressed mainly genes associated with T cell dysfunction such as *TOX2*, *CTLA4*, *HAVCR2* and *ENTPD1* (22).

**Figure 1:**
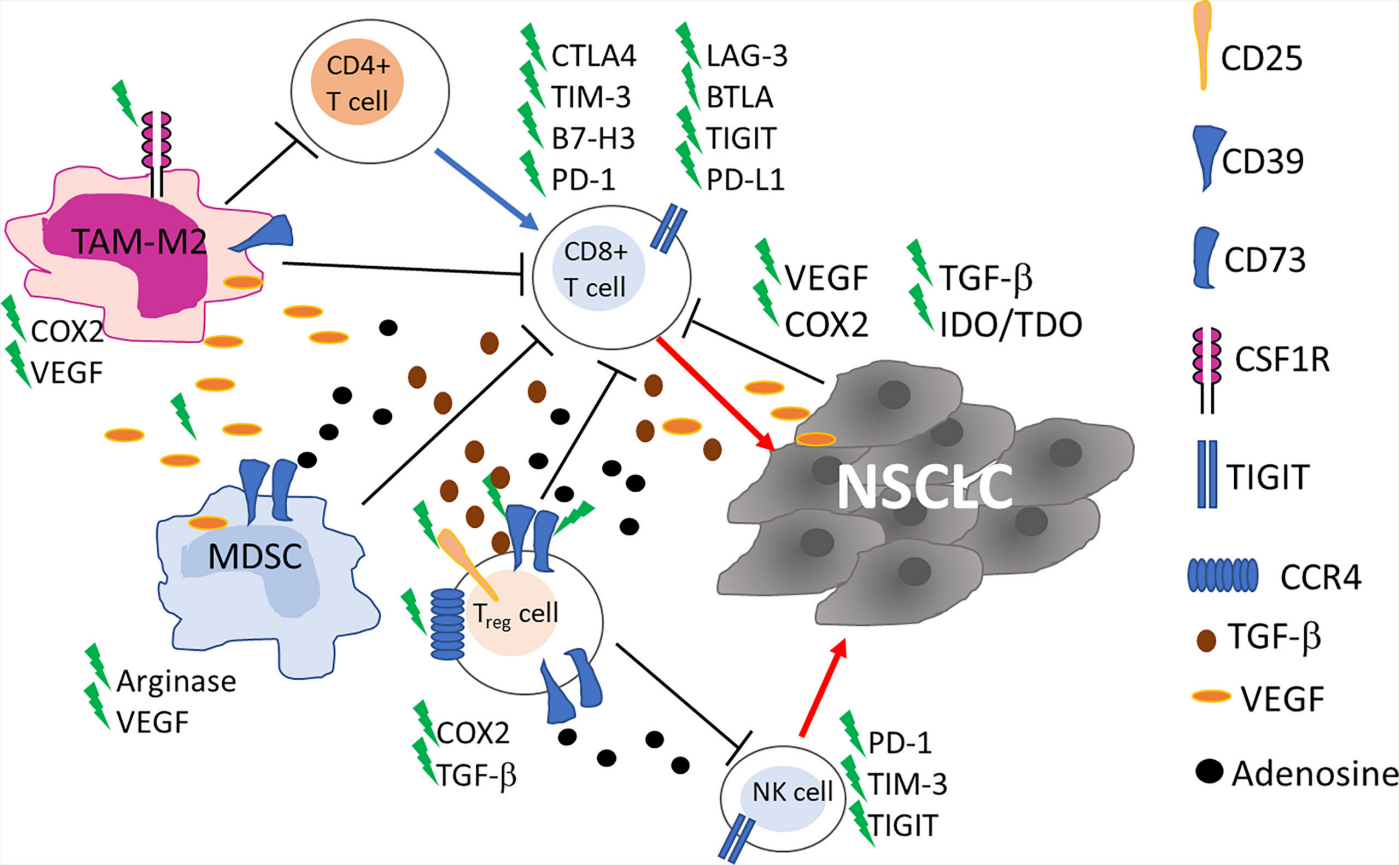
Schematic representation of the main cells in tumor microenvironment involved in NSCLC resistance to ICI. Up-regulation of alternative immune checkpoints on cytotoxic CD8+ T cells impairs recognition and killing of tumor cells. Myeloid derived suppressor cells (MDSC), tumor associated macrophages (TAM)-M2 and CD4+ T Regulatory (Treg) cells through cytokine and soluble factors contribute to the inhibition of the immune responses. Blue and red arrows indicate stimulation and killing, respectively. New targets for on-going clinical trials are highlighted by a green flash.

The presence of alternative immune checkpoint receptors leading to a progressive and profound T-cell exhaustion has been correlated with resistance to ICI (**Figure 1**). Dysfunctional, ‘burned-out’ CD8+ TILs (Ebo) were identified using single-cell mass cytometry and tissue imaging technologies from 25 patients with resectable and 35 patients with advanced NSCLC. Ebo TILs accumulated in the TME, show high proliferation rate and activation markers but produce low amount of interferon-gamma (IFNγ). The presence of these cells expressing high levels of PD-1, TIM-3 and LAG-3 was associated with resistance to cancer immunotherapy (23). The lymphocyte activation gene-3 (LAG-3; CD223) is an inhibitory immune receptor expressed on NK, activated T and B cells and exerts its inhibitory action by binding class II MHC. Regulatory T cells (Treg) cells expressing LAG-3 are more active, while LAG-3 expression in cytotoxic T lymphocytes (CTL) is associated with decreased proliferation and activity. T cell immunoglobulin and mucin domain protein 3 (TIM-3), similarly to LAG-3, is an inhibitory receptor frequently detected upregulated on NSCLC TILs during tumor progression and is associated with an exhausted, burned phenotype of TILs and resistance to ICI (23, 24). In patients with NSCLC PD-1, TIM-3, CTLA-4, LAG-3, and BTLA inhibitory receptors were detected on TILs with a gradual and continuous upregulation during tumor progression, in 24 tumor lesions (24).

In NSCLC the accumulation of NK cells is observed, mainly constituted by non-cytotoxic CD56^bright^CD16^−^ NK cells, a subset endowed with immunoregulatory properties (25, 26). NK cell dysfunction, as well as T cell exhaustion, has also been observed (**Figure 1**). PD-1 is expressed not only on activated T cells, but also on NK cells, and its interaction with anti-PD-1 ICI enhances immune function. In a randomized controlled trial in patients with PD-L1+ NSCLC the combination of *in vitro* expanded allogenic NK cells with anti-PD-1 improved overall survival (OS) and progression-free survival (PFS), compared to single anti-PD-1 treatment, without adverse events associated with NK cell therapy [NCT02843204 (27)]. Killer-cell immunoglobulin-like receptors (KIR) are molecules expressed on the surface of NK cells that, through the engagement of MHC class I ligands expressed on cancer cells, generate inhibitory signals to NK cells. The final result of such interaction is NK cell inactivation (28). He et al. showed that among 11 NSCLC patients treated with nivolumab, 45.5% (n=5) displayed KIR expression in the tumor tissue and in 2 out of 5 increased after treatment with anti-PD-1 ICI (29). However, the authors do not clearly identify NK cells among TILs and analyzed only a small number of patients, thus further studies are needed to point out a real role for KIR in ICI resistance.

### 2.2 Immunosuppression

Frequently, TME is characterized by the presence of cells endowed with immune suppressive activities and an association with resistance to ICI has been reported, in cancer (10, 30, 31). Treg, myeloid derived suppressor cells (MDSC), and tumor associated macrophages (TAM)-M2 through a cytokine network contribute to the inhibition of the immune responses thus inducing immune suppression (**Figure 1**). Treg cells inhibit T cell responses in different ways, and, in general, are associated with poor clinical outcomes in lung cancer patients (32). Recently, an increase in PD-1+Treg has been detected in patients non-responsive to anti-PD-1/PD-L1 ICI in a study evaluating patients with NSCLC (n=27) and other solid cancers. The authors demonstrated that the balance of PD-1 expression between CD8+ T cells and Treg cells in the TME can predict the clinical effectiveness of ICI therapies better than PD-L1 expression or TMB. Anti-PD-1/PD-L1 ICI, while recovering dysfunctional PD-1+CD8+ T cells, may enhance PD-1+ Treg cell-mediated immunosuppression (33). In a previous study on 73 NSCLC patients treated with anti-PD-1/PD-L1 ICI, the density of PD-L1+ Treg in the TME was indicated as an additional prediction biomarker of response to ICI (34), thus Treg warrant consideration as a therapeutic target to augment the clinical efficacy of ICI in lung cancer.

MDSC can affect TME inducing immunosuppression in many different ways: i) producing nitric oxide (NO) and reactive oxygen species (ROS); ii) eliminating key nutrition factors for T cells from the microenvironment, such as L-arginine, and L-tryptophan; iii) interfering with T cells homing and trafficking; iv) inducing up-regulation of checkpoint; v) and releasing immune regulatory molecules, such as adenosine, Vascular endothelial growth factor (VEGF)-alpha and inhibitory cytokine (interleukin (IL)-10) (35). MDSC, like Treg cells, express CD39 and CD73 ectonucleotidases that in tandem convert ATP into adenosine which is considered an important mediator of immune suppression in the TME (36) (**Figure 1**). MDSC expressing CD39 and CD73 were found in tumor tissue of NSCLC patients and positively correlated to disease progression but chemotherapy significantly reduced these cells (37). The role of MDSC in lung cancer outgrowth and ICI therapy has been deeply investigated in preclinical studies in mice (38–40). These studies show that MDSC promote lung cancer metastasis and that their inhibition may overcome resistance to ICI.

The role of TAM has been explored in a cohort of 187 NSCLC patients, mostly treated with ICI. CD163+CD33+PD-L1+ M2-TAM were detected in lesions of patients experiencing hyperprogression. These cells possess an epithelioid morphology (alveolar macrophage-like) and form clusters within neoplastic foci (41). Low CD8+PD-L1+ T cells, and low CD68+CD163+ M2-TAM were predictive for positive response in 33 stage II-IV NSCLC patients treated with ICI (42). By DNA-based quantitative immunofluorescence and confocal microscopy, most PD-L1+ cells are CD68+ macrophages and high cell counts of PD-L1+CD68+ macrophages in the TME has been associated with better OS in 81 patients treated with anti-PD-1 (YTMA404 cohort) (43).

Kargl et al., found that neutrophil content in the TME negatively correlated with the presence of CD8+ and CD4+ T cells and with Th1 and Th17 subsets, but not with Treg cells, implicating a potential immune suppressive role for neutrophils in NSCLC (44, 45). Data from preclinical studies in *IL-17:K-Ras* mutated transgenic mice demonstrated that resistance to anti-PD-1 therapy is abrogated by neutrophil depletion, reconstituting T cell activation (46). The role of neutrophils in the resistance to ICI in NSCLC patients still remains to be addressed.

### 2.3 Angiogenesis

Angiogenesis, with abnormal vasculature is part of TME and is a hallmark of cancer associated with development, proliferation and metastasis (47–49). Vascular endothelial growth factor/vascular endothelial growth factor receptor (VEGF/VEGFR) are a family of proteins that play an essential role in tumor induced angiogenesis promoting vascular permeability by regulating the differentiation, migration, proliferation and survival of microvascular endothelial cells (48). VEGF proteins can inhibit the maturation, differentiation, and antigen presentation of professional Antigen Presenting Cells (APC), DC, NK, and T cells, while improving the suppressive effect of Treg, TAM, and MDSC (**Figure 1**). A comprehensive review on VEGF and its targeting in association with ICI has been published yet in 2021 by Ren et al. (48). Targeting VEGF-A has been exploited in patients to reduce resistance to immunotherapy by combining bevacizumab (anti-VEGFA antibody) with atezolizumab (NCT02366143) and chemotherapy, showing a significant improvement of PFS and OS of patients with metastatic lung cancer (50). This clinical response was independent from PD-L1 expression and genetic alteration status of tumors, and strongly supports a role of angiogenesis in the resistance to ICI.

### 2.4 Tertiary Lymphoid Structures

NSCLC are often associated with the presence of ‘Tertiary Lymphoid Structures’ (TLS). TLS may occur at both the margins and the core of tumors, are spatially well-organized and are composed of T and B cell zones and germinal centers (51). Some authors have correlated the presence of B cells in TLS with favorable outcomes (52–56). In particular, Tang et al. observed an increase in TLS area and B cell proportion within TLS in lung cancer patients with resectable tumors and found a correlation with longer survival rates (56). Since presence and composition of TLS might be influenced by chronic inflammation, TLS from patients who had undergone resection for lung cancer were analyzed, comparing patients with chronic obstructive pulmonary disease (COPD) and those without. Notably, the samples from patients with underlying COPD were characterized by reduced TLS and reduced germinal centers compared to samples from patients without COPD. Follow-up demonstrated poorer survival for patients with fewer TLS, especially among COPD patients (56). These findings imply that chronic inflammation might result in reduced immunological responses against tumorigenesis, but studies on TLS role in ICI resistance need to be pursued for NSCLC patients.

### 2.5 Tumor Driver Mutations and TME

Tumor intrinsic mechanisms, such as specific driver mutations may affect NSCLC resistance to ICI therapy. In particular, D’ Incecco et al. found that PD-1+ tumors are characterized by *KRAS* mutations, whereas PD-L1+ tumors are mainly *EGFR* mutated (57). *EGFR* mutated NSCLC exhibited reduced CD8+ lymphocyte infiltration, while *KRAS* mutant displayed higher CD8+ T cells, as detected using tissue microarray (58). By single-cell RNA sequencing on NSCLC tissue harboring *EGFR* mutation, myeloid and T cells, mainly exhausted, and Treg, were the most abundant immune cells identified (59). The reasons for the weak response of *EGFR*-positive NSCLC patients to ICI are still not fully understood. *EGFR* mutated tumors have lower somatic mutations and number of neoantigens (60), display an uninflamed TME, which may explain the poor efficacy of ICI compared to *EGFR*-wild type (61). The role of *EGFR* mutation on the upregulation of PD-L1 expression is still controversial (62). *STK11/LKB1* alterations confer to NSCLC resistance to PD-1 blockade, in a study conducted on 66 patients with PD-L1+ tumors receiving anti-PD-1/PD-L1 therapy (63). In particular, *STK11/LKB1* alterations were frequently associated to *KRAS* mutations and with low TILs, reduced PD-L1 expression and high TMB (63). In a genetic engineered mouse model bearing *KRAS* and *STK11/LKB1* mutations a massive recruitment of immunosuppressive neutrophils and increase in the expression of exhaustion marker on T cells was detected (64).

Resistance to ICI may also be driven by loss of antigen presentation occurring in antigen presenting cells or cancer cells within the TME, and is frequently associated with acquired genetic mutations, such as loss of heterozygosity (LOH) in HLA loci, mutation of HLA genes, and modulation of HLA gene expression (65, 66).

Recently, Bagaev et al. have developed a multi-omics and robust analytical platform to classify, reconstruct, and visualize the entire tumor composition (67). They took into consideration genomic and transcriptomic analyses that evaluate the tumor (mutations of DNA repair genes, and cell cycle regulation) and the TME (the major functional components and immune, stromal, and other cellular populations) as a whole for different cancers. They defined four distinct TME subtypes predictive of response to immunotherapy [Immune-Enriched, Fibrotic (IE/F); Immune-Enriched, Non Fibrotic (IE); Fibrotic (F); Depleted (D)] based on melanoma that were conserved across at least 20 additional cancers, including lung cancer [n=27 (67)]. Subtype IE had significantly longer OS and PFS compared to F and D, with F being the worst, in melanoma. Lung cancer patients with TME subtype IE demonstrated the longest OS. Genetic alterations, such as *EGFR* in lung cancer, were associated to F and D TME subtypes.

## 3 Immune Related Signatures

In the last decade great efforts have been made to identify reliable predictive TME-based signatures for lung cancer immunotherapy. Currently, one of the most powerful prognostic tools in oncology is “immunoscore” (IS) based on the numbering of T lymphocytes within the tumor (68). This tool is a digital tumor tissue-based test that estimates patient’s prognosis on immune cell infiltration (*i.e.*, CD3/CD45RO, CD3/CD8, or CD8/CD45RO). Specifically, IS measures the subpopulations of T cells in the center and periphery of the tumor and provides a score ranging from IS 0 with a low density of immune cells to IS 4 with a high density in both regions. This test, initially validated on colorectal cancers (68), has shown great promise as a supplement to the classification of lymph node metastases (TNMs) in a number of cancers, including NSCLC (69). In particular, numerous studies have shown that a high IS score correlates with better survival (70–73). In addition, CD8+ TIL has also been described as a powerful biomarker in discriminating patients with a significantly longer PFS after ICI treatment; this association was strengthened when IS was integrated with tumor PD-L1 expression, suggesting that the combination of these markers could be a reliable biomarker for immunotherapy (74). Gene signatures, an alternative approach to characterize the TME on the transcriptomic profiling, have recently gained a great interest in the scientific community. The TME signature consists of lists of genes indicative of the presence of a given population of immune/stromal cells and/or descriptive of a particular state of TME-cell activation.

With the advent of high-throughput technologies (*i.e.* microarray and more recently RNA seq) capable of screening the whole transcriptome of the tumor bulk, an increasing number of computational algorithms have been developed for the prediction of non-cancer cell infiltration (**Table 1**) (75, 79, 82, 85, 86, 88, 89).

**Table 1:** Current state-of-art computational tools.

Name	Year	Type	Output	Web-server	Code
CIBERSORT (75)	2015	DB	Fractions of the immune cell-types defined by the signature matrix provided in input and corresponding p-value	https://cibersort.stanford.edu/(registration required)	External R package: https://github.com/icbi-lab/immunedeconv
CIBERSORTx (76, 77)	2019	DB	(i) custom gene signature matrix computed from scRNA-seq or bulk sorted RNA-seq data (ii) cell type proportion inferred from GEPs by using the computed (or provided) gene signature matrix (iii) cell-type specific GEPs.	https://cibersortx.stanford.edu (registration required)	N.A.
EPIC (78)	2017	DB	Fractions of (i) individual non-malignant cell-types for which a GEP is provided (ii) all the other non-characterized (cancer) cell types grouped together. The package provides reference GEPs for B, CD4 T, CD8 T, NK, CAFs, Endothelial, Macrophages, Monocytes, Neutrophils.	http://epic.gfellerlab.org	R package: https://github.com/GfellerLab/EPIC
ESTIMATE (79)	2013	SB	Two scores representing the level of immune and stromal cells. A derived level of tumor purity.	N.A.	R package: https://bioinformatics.mdanderson.org/estimate/
Gene signature of infiltrating Leukocytes (80)	2017	SB	60 GS for 14 immune cell types (B, CD45, Cytotoxic, Exhausted CD8, Macrophages, Mast cells, Neutrophils, NK, NK CD56dim, T, Th1, Treg, CD8, CD4) derived testing gene signatures from the literature.	N.A.	R code for reproducing the analysis as supplementary material of the paper.
Immunophenoscore (81)	2017	SB	782 GS for 28 immune cell types (T, Tcm, Tem, activated, central memory, CD4+, CD8+, gamma delta T, Th1, Th2, Th17, Treg, Tfh, activated, immature, and memory B, machrophage, monocytes, mast cells, esosinophils, neutophils, acitvated, monocytes, and immature DC, NK, NKT, MDSC). An aggregate score, tarmed immunophenoscore, quantifying tumour immunogenicity.	https://tcia.at	R package: https://github.com/icbi-lab/Immunophenogram
MCP-Counter (82)	2016	SB	Abundance score for 8 immune cell types (T cells, CD8+ T cells, NK cells, cytotoxic lymphocytes, B cell lineage, monocytic lineage cells, myeloid dendritic cells, and neutrophils) and 2 stromal cell types (endothelial cells and fibroblasts)	http://134.157.229.105:3838/webMCP/	R package: https://github.com/ebecht/MCPcounter
QuanTIseq (83)	2019	DB	Absolute fractions for 10 immune cell types (B cells, M1 and M2 macrophages, monocytes, neutrophils, NK cells, CD4+ T cells, CD8+ T cells, Treg cells, and myeloid dendritic cells) and abundance of the remaining uncharacterized cells.	N.A.	Pipeline: http://icbi.at/quantiseq (Raw FASTQ data allowed) R package: https://bioconductor.org/packages/release/bioc/html/quantiseqr.html
TIP (84)	2018	Both	(I) 23 immune activity score computed based on 178 signature genes. This score quantifies the activity status of the 7-step immunity cell-cycle. (II) Relative proportion of tumor-infiltrating immune cells computed by CIBERSORT. If microarray GEPs are provided the original signature matrix with 22 cell-types is used; if RNA-seq data are provided a dedicated signature matrix with 24 cell-types is used.	http://biocc.hrbmu.edu.cn/TIP/	R package: https://github.com/dengchunyu/TIP
TIMER (85, 86)	2016	DB	Relative abundance of 6 immune cell types: B cells, CD4 T cells, CD8 T cells, neutrophils, macrophages, dendritic cells.	https://cistrome.shinyapps.io/timer/	R package: http://cistrome.org/TIMER/download.html
TIMER 2.0 (87)	2020	Both	Results and comparison from TIMER, xCell, MCP-counter, CIBERSORT, EPIC, quanTIseq	http://timer.cistrome.org/	External R package: https://github.com/icbi-lab/immunedeconv
Xcell (88)	2017	SB	GS score for 64 immune and stroma cell types corrected for spillover effects.	https://xcell.ucsf.edu/	R package: https://github.com/dviraran/xCell

Two groups of methods exist namely signature-based (SB) and deconvolution-based (DB) approaches. SB approaches identify a set of genes whose expression is characteristic of a specific type of cell. Then, a score is defined to quantify the abundance of each cell type based on the expression of the corresponding signature genes. DB approaches formulate the problem as a mathematical deconvolution, that is the tissue gene expression profile (GEP) is written as the weighted sum of precomputed typical expression profiles of the considered cell-types. The unknown weights are then estimated by using a proper regression technique. For each tool we report: the year of publication of the paper; the method DB and SB approaches; the type of cells for which abundance is computed; possible available web-server and/or open-source package implementing the method. GS, gene signature; NA, not available.

Despite each algorithm varies in terms of computational approach, the output consists of a score based on tumor-infiltrating immune and/or stromal cells, allowing a better comprehension of the mechanisms underlying cancer immunity and their potential role in the response to ICI. The output scores consist of TME signature allowing a comprehension of the intra tumoral heterogeneity as well as the inter-sample comparisons. Among the most relevant studies on the evaluation of the cancer immune landscape using the gene expression profile, the Cancer Genome Atlas Network project deserves to be mentioned (90). The consortium performed a large immuno-genomic study of over 10,000 tumors across 33 cancers by integrating the mRNA expression profile with DNA copy number and mutational status. Then, applying a combination of computational algorithms, the authors characterized the TME in six major immune subtypes defined as follows: 1) wound healing, 2) IFN-dominant, 3) inflammatory, 4) lymphocyte depletion, 5) immunologically silent, and 6) TGF-β dominant. Lung neoplasms were mainly enriched in the first three subtypes; in particular, squamous cell carcinomas (SCCs) showed an enrichment of ‘wound healing’ (defined by high angiogenic gene expression, elevated proliferation rate and Th2 cell bias for adaptive immune infiltrate) and ‘IFN-dominant’ (depicted by high M1/M2 macrophage ratio polarization and a strong CD8 signal such as a high diversity TCR) subtypes. In contrast, lung adenocarcinoma (ADC) showed greater enrichment of ‘INF dominant’ and ‘inflammatory’ (characterized by elevated Th17 and Th1 genes, low/moderate tumor cell proliferation, and low levels of aneuploidy) subtypes. A similar extensive bioinformatic strategy was also performed by Charoentong et al. who, by integrating DNA and RNA data over 8,000 patients across 20 solid cancers, defined an immunophenoscore, able to discriminate patients more responsive to ICI (81). In particular, the predictive score provides information on some relevant immunogenomic characteristics such as TIL composition, cancer antigen profiles and tumor heterogeneity. Another pan-cancer study that examined the TME gene profile aimed at predicting clinical response to PD-1 blockade, was performed by Ayers in 2017 (91). The authors, starting from a small pilot study including 19 patients with metastatic melanoma undergoing anti-PD-1 ICI, profiled the expression of 680 tumor and immune genes using the digital platform NanoString nCounter (91, 92). Through a rigorous multi-step validation, they defined an 18-gene score, named ‘Tumor Inflammation Signature’ (TIS), that included genes linked to cytotoxic cells, antigen presentation, and IFNγ activity. More recently, the prognostic value of the TIS score was also evaluated in the 9,083 tumor gene expression profiles downloaded from the Cancer Genome Atlas (TCGA) database (980 from lung cancers) (93). As already reported in the previous study, tumors with known clinical sensitivity to ICI such as NSCLC, showed generally higher TIS scores. In addition, the TIS score showed a stronger prediction for identifying patients with clinical sensitivity to ICI than TMB status, especially in tumors with low TMB variability, such as SCC. In the wake of these intriguing findings, an exponential number of studies have profiled TME genes on lung cancers by identifying highly specific and accurate signatures capable of predicting molecular subtypes more sensitive to anti-PD-L1/PD1-based therapies (94–97). For example, Higgs et al. identified an IFNγ signature, focused on 4 genes already included in the previous TIS such as *IFNγ, LAG3, CXCL9* and *PD-L1* (94). IFNγ-positive signature patients showed higher overall response rates and better PFS and OS with the anti-PD-L1 durvalumab, regardless of tissue PD-L1 status. In addition, several studies downloaded RNA datasets from public databases and using mathematical models each score was then tested in independent validation sets to improve prediction performance (97–101). Chaoqi Zhang et al. using more than 1,500 RNA data from ADC tumors, tested 60 costimulatory molecule genes on 502 cases. Then, applying a step-wise method, they filtered the combination of 5 genes which was validated on ten independent sets. The costimulatory molecule 5 gene-based signature identified two risk groups with distinct inflammatory profiles and immune infiltrate, through a computational method. ‘High-risk’ patients had a significantly higher proportion of activated NK cells, DC, neutrophils, macrophages M0, resting DC, and Treg. ‘Low-risk’ patients had a high proportion of memory B cells, resting CD4 memory T cells, and gamma delta T cells. According to the profiles, the authors indirectly predicted that high-risk patients could benefit from immunotherapy (98).

### 3.1 Novel Emerging Signature

Despite the impressive results, the tissue-based immune signatures require the collection of representative tumor specimens and can therefore be limited by inadequate samples or by intra-tumoral heterogeneity, commonly described in NSCLC. To date, radiomics represents one of the most promising across the emerging predictive biomarkers for ICI. Radiomics is a high-throughput extraction of features from medical images using computer algorithms, aimed at providing quantitative information on tissue composition that otherwise cannot be detected through simple observation (102, 103). Ideally, radiomics can be considered as a virtual biopsy with the advantage of being a totally non-invasive tool, which allows the evaluation of the tumor and its microenvironment, the characterization of intra-tumoral heterogeneity and a dynamic monitoring. One of the first application of the radiomics in the characterization of molecular heterogeneity of lung cancers dated in 2012. The authors compared images from preoperative computed tomography (CT) and Positron Emission Tomography/Computed Tomography (PET/CT) from a cohort of 26 NSCLC patients with tissue gene expression profiles (radiogenomics) identifying significant correlations (104). In the last decade, a growing number of studies have investigated the potential clinical utility of radiomic features (RFs) providing radiomic-based signatures for precision diagnosis as well as the prediction of gene mutations (105–107). In addition, the radiomic approach has also been applied to decipher lung TME (108, 109). Recently, Chen and colleagues, applying the least absolute shrinkage and selection operator (LASSO) and logistic regression to CT images from 120 patients, extracted 462 RFs. The combined model, including RFs, clinical and morphological data, showed an optimal prediction power for PD-L1 expression levels and TMB status (110). A number of studies also reported image-based signatures predictive to ICI response or outcome (111, 112). Very recently, Yang and colleagues used pretreatment CT images, from 92 patients treated with an ICI, to select 88 RFs. Then, the authors, developed two nomogram-based models, integrating RFs with clinical pathological characteristics and demonstrated good performances in identifying patients with a durable response and a longer PFS (113). In another retrospective study, Khorrami et al. applying a machine learning approach, compared the delta radiomic texture (DelRADx) of CT patterns both in the tumor and peritumoral regions between the baseline and the post-treatment scans of 139 advanced patients receiving ICI. The combination of eight identified DelRADx features were predictive of response to ICI therapy and of OS (114). Similarly, a new algorithm ‘TMB radiomic biomarker’ (TMBRB) combining deep learning technology to CT images from 327 NSCLC patients distinguished tumors with a High-TMB versus a Low-TMB value. TMBRB, in a cohort of 123 patients treated with an ICI resulted an optimal predictor in terms of both OS (HR: 2.33, 95%CI: 1.14 to 4.77) and PFS (HR: 1.90, 95%CI: 1.14 to 3.19) (115). Recently, DelRADx features resulted predictive of response to ICI therapy, prognostic of improved OS, and correlated with TIL density (114).

## 4 Immunobiology Of Lung Cancer

Several lines of evidence highlight the roles of both innate and adaptive immune components in the elimination phase of cancer immunoediting process. The adaptive branch of the immune system has been demonstrated as the prominent mechanism able to eliminate cancer cells through the recognition of tumor antigen in the context of MHC complex (116).

Tumor associated antigens (TAA) overexpressed in lung cancer are MUC-1, CEA, NY-ESO, MAGE-A3 (117–119). Due to their expression in normal cells, these antigens are considered less immunogenic and more likely to induce tolerance, furthermore tumors expressing these antigens seem less responsive to ICI.

Conversely, tumor specific antigens (TSA) are unique to tumor cells and should result from non-synonymous somatic mutations thus represent the ideal antigens for cellular immunotherapy (120, 121). Several reports have demonstrated that tumors with a high TMB, like NSCLC, possess a high number of neoantigens. Among the various somatic mutations noted, some occur in driver genes including in *TP53, KRAS, CDKN2A, ARID1A, NOTCH1, MYC, SMARCA4* and *RB1* (122, 123). Neoantigens can be recognized by TILs. Accordingly, neoantigen density has been shown to correlate with a favorable prognosis and higher CTL content (124) as well as, with benefit from ICI (125). Despite being extremely challenging, neoantigen-specific cells have been successfully identified in NSCLC patients by using the Mutation Associated NeoAntigen Functional Expansion of Specific T-cells (MANAFEST) platform (126). CTL specific for peptides derived from oncogenic driver mutations such as *TP53* R248L (22), or *BRAF* N581I (127) have been found.

Cancer vaccines aim at boosting T cell and B cell-mediated response against TAA or TSA. Several clinical trials are currently evaluating different vaccines in lung cancer patients and specific target antigens (*e.g*. MAGE-A3, CEA, mesothelin, RAS, NY-ESO-1, telomerase, WT1), as well as immunomodulatory enzymes such as Indoleamine 2,3-dioxygenase (IDO) and Arginase-1 (119, 128). Interestingly, some of these cancer vaccines have been recently administered also in combination with ICI in phase I/II studies (i.e. NCT04908111, NCT02879760, NCT03562871), even if no data regarding effectiveness has been released yet.

Tumor neoantigens are highly specific to tumors of an individual patient and not expressed on normal cells, thus able to evoke robust tumor-specific T cell responses (129). To date, several clinical trials are ongoing investigating personalized neoantigen-based vaccines alone or in combination with anti-PD-1, -PD-L1 and/or -CTLA-4 antibodies in various tumor types, comprising NSCLC (130). Neoantigens can be identified by multiple bioinformatic technologies, mainly based on whole-exome sequencing computational algorithms for antigen prediction. Personalized vaccines are being developed and employed in different formulations, such as synthetic long peptide (SLP), DNA, RNA, DC-based, and associated to viral and bacterial vectors (131). Recently, data from a phase Ib trial of personalized neoantigen therapy (NEO-PV-01, NCT02897765) plus nivolumab in patients with Advanced Melanoma, NSCLC (n=18), or Bladder Cancer was released, demonstrating that this type of regimen was safe and did not lead to treatment-related serious adverse events. In addition, the data demonstrated that the vaccine was able to trigger an effective T cell response against neoantigens in all vaccinated patients. Interestingly, the vaccine evoked a T cell response also to neoantigens not included in the vaccine formulation (epitope spread) (132).

Targeting of tumor antigens has been also pursued by adoptive transfer of tumor-reactive T Cells (ACT). Upon isolation from the patient, natural or *in-vitro*-modified T cells are expanded ex vivo and reintroduced into the patient to enhance T cell responses and kill tumor cells. ACT therapies include the adoptive transfer of TILs, or of engineered T cells that possess retargeted specificity and higher affinities for tumor antigens, such as engineered affinity-enhanced αβTCR or chimeric antigen receptors (CAR). Compared to vaccine-based strategies, ACT provides patient with already competent effector cells, thus overcoming the requirement of T-cell priming in patients who are often immune compromised and tolerant to cancer antigens. Current strategies for targeting advanced NSCLC include adoptive transfer of engineered T cells directed against specific TAA, such as NY-ESO-1/LAGE-1, also in combination with ICI (NCT03709706), as well as personalized adoptive cell therapy where neoantigen-specific T cells from individual tumors are identified, expanded ex vivo, and then re-injected in patients (NCT04596033). Despite being very promising, TCR-based ACT may suffer from certain disadvantages. αβTCR-based targeting approaches remain susceptible to tumor escape arising through immunoediting processes that select tumor clones unable to present antigens due to impairment in MHC-class I expression or to interference with antigen presentation (66, 133). More recently, by analyzing next-generation sequencing data derived from previous early-stage NSCLC and matched brain metastases, McGranahan et al. found that 40% of early-stage NSCLC displayed LOH and that metastases had an even higher prevalence of such genetic alteration. Interestingly, HLA-LOH in metastasis was associated with an elevated non-synonymous mutation rate, suggesting LOH as an immune escape mechanism that prevents presentation of neoantigens (134). To circumvent the loss of MHC and antigen presentation, transduction of patient’s T cells with chimeric antigen receptors (CAR) recognizing intact cell surface proteins represents an alternative approach to redirect T cell specificity. However, exploitation of CAR T cell technology in solid tumors still presents many hurdles. In order to overcome these limitations, CAR-T cells have now been engineered to enhance tumor infiltration, induce the remodeling of the TME and endogenous immune response, and disrupt immunosuppressive axes (135). This is the case, for example, of an early phase I clinical trial which exploits the possibility to use CAR-T cells directed against mesothelin (MSLN) further engineered to secrete, locally, anti-PD-1 antibodies in NSCLC and mesothelioma patients [NCT04489862 (136)]. The possibility to target EGFR expressed by NSCLC cells has been also investigated by the use of anti-EGFR CAR T, further modified to express C-X-C Chemokine receptor type 5 (CXCR5), in a phase I clinical study (NCT04153799). Although these trials estimate to recruit small numbers of patients, results will be very important to define the safety and the toxicity of these approaches.

Besides T cells, also NK cells are suitable for engineering with CAR constructs. NK cells equipped with CAR have demonstrated safety, such as a lack or minimal cytokine release syndrome and neurotoxicity, in an autologous setting. CAR-NK cells can also kill targets in a CAR-independent manner (137). Clinical trials evaluating CAR-NK cells for the treatment of solid tumors have been started also in NSCLC (NCT02839954). This phase I/II trial uses CAR-NK cells specific for MUC-1 antigen expressed by different cancers, including NSCLC. Because activated NK cells, similarly to T cells, can express immune checkpoint molecules (*e.g.*, PD-1, LAG-3, and TIM-3) that might inhibit NK anti-tumor responses their blockade with ICI could be envisaged in order to reinvigorate cytotoxic activity (138–140).

## 5 Novel Immunotherapeutic Approaches

Since TME is able to greatly influence immune response through complex pathways, its components represent promising targets for investigational agents. Current immune-oncology research is focusing on the association of “classic”, acknowledged ICI, such as anti-PD-1/PD-L1 and anti-CTLA-4 agents, with investigational compounds, either directed at TME molecules or at newly discovered immune checkpoints. The aim of these novel combinations is to overcome the resistance to ICI and hence improve survival of NSCLC patients. The currently available information on these agents have been reported in the following sub-sections. Notably, as most clinical studies are still ongoing, they have been resumed in **Table 2**.

**Table 2:** Ongoing clinical trials.

anti-LAG3 and ICI							
NCT number	Trial	Status	Phase	Total Estimated enrollment	Investigator	First Submitted Date	Last Update Posted Date
**NCT03625323**	Combination Study With Soluble LAG-3 Fusion Protein Eftilagimod Alpha (IMP321) and Pembrolizumab in Patients With Previously Untreated Unresectable or Metastatic NSCLC, or Recurrent PD-X Refractory NSCLC or With Recurrent or Metastatic HNSCC (TACTI-002) - TACTI-002 Keynote-PN798 (Other Identifier: Merck Sharp & Dohme Corp)	Recruiting	Phase II	183	Frederic Triebel	August 10, 2018	April 9, 2021
**NCT04140500**	Dose Escalation Study of a PD1-LAG3 Bispecific Antibody in Patients With Advanced and/or Metastatic Solid Tumors	Recruiting	Phase I	320	Reference Study ID: NP41300 www.roche.com/about_roche/roche_worldwide.htm	October 28, 2019	July 22, 2021
**NCT03219268**	A Study of MGD013 in Patients With Unresectable or Metastatic Neoplasms	Active, not recruiting	Phase I	353	Bradley Sumrow, MD MacroGenics	July 17, 2017	August 9, 2021
**NCT03250832**	Study of TSR-033 With an Anti-programmed Cell Death-1 Receptor (PD-1) in Participants With Advanced Solid Tumors (CITRINO)	Active, not recruiting	Phase I	111	GSK Clinical Trials Glaxo SmithKlin	August 16, 2017	May 18, 2021
**NCT04641871**	Sym021 in Combination With Either Sym022 or Sym023 in Patients With Advanced Solid Tumor Malignancies	Active, not recruiting	Phase I	200	Nehal Lakhani, MD START Midwest	November 24, 2020	May 14, 2021
**NCT03849469**	A Study of XmAb^®^22841 Monotherapy & in Combination w/Pembrolizumab in Subjects w/Selected Advanced Solid Tumors (DUET-4)	Recruiting	Phase I	242	Benjamin Thompson, MD, PhD Xencor, Inc.	February 21, 2019	May 5, 2021
**NCT04623775**	A Study of Relatlimab Plus Nivolumab in Combination With Chemotherapy vs. Nivolumab in Combination With Chemotherapy as First Line Treatment for Participants With Stage IV or Recurrent Non-small Cell Lung Cancer (NSCLC)	Recruiting	Phase II	520	Bristol-Myers-Squibb	November 10, 2020	August 25, 2021
***anti-TIM-3 and ICI***							
**NCT03708328**	A Dose Escalation and Expansion Study of RO7121661, a PD-1/TIM-3 Bispecific Antibody, in Participants With Advanced and/or Metastatic Solid Tumors	Recruiting	Phase I	280	Clinical Trials Hoffmann-La Roche	October 17, 2018	July 19, 2021
**NCT04931654**	A Study to Assess the Safety and Efficacy of AZD7789 in Participants With Advanced or Metastatic Solid Cancer	Not yet recruiting	Phase I	81	AstraZeneca	June 18, 2021	July 16, 2021
**NCT03652077**	A Safety and Tolerability Study of INCAGN02390 in Select Advanced Malignancies	Active, not recruiting	Phase I	40	John Janik, MD Incyte Corporation	August 29, 2018	March 17, 2021
**NCT04641871**	Sym021 in Combination With Either Sym022 or Sym023 in Patients With Advanced Solid Tumor Malignancies	Active, not recruiting	Phase I	200	Nehal Lakhani, MD START Midwest	November 24, 2020	May 14, 2021
**NCT02817633**	A Study of TSR-022 in Participants With Advanced Solid Tumors (AMBER)	Recruiting	Phase I	369	GSK Clinical Trials GlaxoSmithKline	June 29, 2016	June 8, 2021
**NCT03307785**	Previous Study | Return to List | Next Study Study of Niraparib, TSR-022, Bevacizumab, and Platinum-Based Doublet Chemotherapy in Combination With TSR-042	Active, not recruiting Has results	Phase I	58	GSK Clinical Trials GlaxoSmithKline	October 12, 2017	May 10, 2021
**NCT02608268**	Phase I-Ib/II Study of MBG453 as Single Agent and in Combination With PDR001 in Patients With Advanced Malignancies	Active, not recruiting	Phase I Phase II	252	Novartis Pharmaceuticals	November 18, 2015	July 19, 2021
**NCT03099109**	A Study of LY3321367 Alone or With LY3300054 in Participants With Advanced Relapsed/Refractory Solid Tumors	Active, not recruiting	Phase I	275	Eli Lilly and Company	April 12, 2017	September 5, 2021
***anti-B7-H3 and ICI***							
**NCT02475213**	Safety Study of Enoblituzumab (MGA271) in Combination With Pembrolizumab or MGA012 in Refractory Cancer	Active, not recruiting	Phase I	145	Stacie Goldberg, M.D. MacroGenics	June 18, 2015	April 14, 2021
**NCT02381314**	Safety Study of Enoblituzumab (MGA271) in Combination With Ipilimumab in Refractory Cancer	Completed	Phase I	24	Stacie Goldberg, M.D. MacroGenics	March 6, 2015	March 25, 2019
**NCT03729596**	MGC018 With or Without MGA012 in Advanced Solid Tumors	Recruiting	Phase I Phase 2	182	Chet Bohac,PharmD MD MSc MacroGenics	November 2, 2018	April 28, 2021
***anti-TIGIT and ICI***							
**NCT04995523**	A Study to Assess the Safety and Efficacy of AZD2936 in Participants With Advanced or Metastatic Non-small Cell Lung Cancer (NSCLC) (ARTEMIDE-01)	Not yet recruiting	Phase I Phase II	147	AstraZeneca	August 9, 2021	August 9, 2021
**NCT04952597**	Study of Ociperlimab Plus Tislelizumab Plus Chemoradiotherapy in Participants With Untreated Limited-Stage Small Cell Lung Cancer	Recruiting	Phase II	120	BeiGene	July 7, 2021	July 30, 2021
**NCT04746924**	A Study of Ociperlimab With Tislelizumab Compared to Pembrolizumab in Participants With Untreated Lung Cancer	Recruiting	Phase III	605	Mark Socinski, MD Advent Health Orlando	February 10, 2021	June 14, 2021
**NCT04672356**	A Study to Evaluate the Safety, Tolerability and Efficacy of IBI939 in Combination With Sintilimab in Patients With Advanced Lung Cancer	Recruiting	Phase I	20	Ying Cheng Jilin Province Cancer Hospital	December 17, 2020	February 21, 2021
**NCT04294810**	A Study of Tiragolumab in Combination With Atezolizumab Compared With Placebo in Combination With Atezolizumab in Patients With Previously Untreated Locally Advanced Unresectable or Metastatic PD-L1-Selected Non-Small Cell Lung Cancer (SKYSCRAPER-01)	Recruiting	Phase III	560	Hoffmann-La Roche	March 4, 2020	July 20, 2021
**NCT04791839**	Safety and Efficacy of Zimberelimab (AB122) in Combination With Domvanalimab (AB154) and Etrumadenant (AB928) in Patients With Previously Treated Non-Small Cell Lung Cancer	Recruiting	Phase II	30	Daniel MorgenszternM.D. Washington University School of Medicine	March 10, 2021	August 11, 2021
**NCT04672369**	A Study to Evaluate the Efficacy of IBI939 in Combination With Sintilimab in Patients With Advanced NSCLC	Not yet recruiting	Phase I	42	Ying Cheng Jilin Province Cancer Hospital	December 17, 2020	December 17, 2020
**NCT04866017**	Tislelizumab Plus BGB-A1217 Versus Tislelizumab Versus Durvalumab When Co-administered With Concurrent Chemoradiotherapy (cCRT) in Lung Cancer	Recruiting	Phase III	900	Yalan Yang, MD BeiGene	April 29, 2021	July 1, 2021
***anti-KIRs and ICI***							
**NCT03347123**	A Study of Epacadostat and Nivolumab in Combination With Immune Therapies in Subjects With Advanced or Metastatic Malignancies (ECHO-208)	Completed	Phase I Phase II	11	Incyte Corporation	November 20, 2017	April 19, 2021
***anti-NKG2A and ICI***							
**NCT03822351**	Durvalumab Alone or in Combination With Novel Agents in Subjects With NSCLC (COAST)	Active, not recruiting	Phase II	189	AstraZeneca	December 19, 2018	August 4, 2021
***Targeting immune suppression and ICI***							
**NCT03621982**	Study of ADCT-301 in Patients With Selected Advanced Solid Tumors	Recruiting	Phase I	95	ADC Therapeutics	August 9, 2018	July 13, 2021
**NCT04396535**	Docetaxel With or Without Bintrafusp Alfa for the Treatment of Advanced Non-small Cell Lung Cancer	Recruiting	Phase II	80	Alex A Adjei Mayo Clinic in Rochester	May 20, 2020	May 4, 2021
**NCT02903914**	Arginase Inhibitor INCB001158 as a Single Agent and in Combination With Immune Checkpoint Therapy in Patients With Advanced/Metastatic Solid Tumors	Active, not recruiting	Phase I Phase II	260	Sven Gogov, MD Incyte Corporation	September 16, 2016	March 23, 2021
**NCT03322540**	Pembrolizumab Plus Epacadostat vs Pembrolizumab Plus Placebo in Metastatic Non-Small Cell Lung Cancer (KEYNOTE-654-05/ECHO-305-05)	Completed	Phase II	154	Lance Leopold, MD Incyte Corporation	October 26, 2017	January 6, 2021
**NCT03343613**	A Study of LY3381916 Alone or in Combination With LY3300054 in Participants With Solid Tumors	Terminated (Study terminated due to strategic business decision by Eli Lilly and Company.)	Phase I	60	Eli Lilly and Company	November 17, 2017	June 9, 2020
**NCT02298153**	A Study of Atezolizumab (MPDL3280A) in Combination With Epacadostat (INCB024360) in Subjects With Previously Treated Stage IIIB or Stage IV Non-Small Cell Lung Cancer and Previously Treated Stage IV Urothelial Carcinoma (ECHO-110)	Terminated (Study halted prematurely and will not resume; participants are no longer being examined or receiving intervention.)	Phase I	29	Hiroomi Tada, MD Incyte Corporation	November 21, 2014	December 11, 2017
**NCT03562871**	IO102 With Pembrolizumab, With or Without Chemotherapy, as First-line Treatment of Metastatic NSCLC	Active, not recruiting	Phase I Phase II	108	James Spicer, MD ProfGuy’s Hospital	June 20, 2018	May 19, 2021
**NCT03502330**	APX005M With Nivolumab and Cabiralizumab in Advanced Melanoma, Non-small Cell Lung Cancer or Renal Cell Carcinoma	Recruiting	Phase I	120	Harriet Kluger, MD Yale University	April 18, 2018	December 22, 2020
**NCT04306900**	TTX-030 in Combination With Immunotherapy and/or Chemotherapy in Subjects With Advanced Cancers	Recruiting	Phase I	185	Trishula Therapeutics, Inc.	March 13, 2020	September 30, 2021
**NCT03884556**	TTX-030 Single Agent and in Combination With Immunotherapy or Chemotherapy for Patients With Advanced Cancers	Recruiting	Phase I	100	Trishula Therapeutics, Inc.	March 1, 2019	May 3, 2021
***Targeting Angiogenesis and ICI***							
**NCT04900363**	A Trial of AK112 (PD-1/VEGF Bispecific Antibody) in Patients With NSCLC	Recruiting	Phase I/II	360	Caicun Zhou, MD	May 25, 2021	May 25, 2021
***Targeting cancer cell death and ICI***							
**NCT03775486**	Study of Durvalumab+ Olaparib or Durvalumab After Treatment With Durvalumab and Chemotherapy in Patients With Lung Cancer (ORION)	Active, not recruiting	Phase II	401	Myung-Ju Ahn, MD	December 14, 2018	April 28, 2020
**NCT03976323**	Study of Pembrolizumab With Maintenance Olaparib or Maintenance Pemetrexed in First-line (1L) Metastatic Nonsquamous Non-Small-Cell Lung Cancer (NSCLC) (MK-7339-006, KEYLYNK-006)	Active, not recruiting	Phase III	792	Merck Sharp & Dohme Corp.	June 6, 2019	May 18, 2021
**NCT03976362**	A Study of Pembrolizumab (MK-3475) With or Without Maintenance Olaparib in First-line Metastatic Squamous Non-small Cell Lung Cancer (NSCLC, MK-7339-008/KEYLYNK-008)	Recruiting	Phase III	735	Merck Sharp & Dohme Corp.	June 6, 2019	October 1, 2021
**NCT03307785**	Study of Niraparib, TSR-022, Bevacizumab, and Platinum-Based Doublet Chemotherapy in Combination With TSR-042	Active, not recruiting	Phase I	58	Tesaro, Inc.	October 12, 2017	May 10, 2021

### 5.1 Targeting Emerging Immune Checkpoints

Recently, several novel immune checkpoints with potential therapeutic have been identified, and the most promising molecules appear to be LAG-3, TIM-3, B7-H3, and TIGIT.

LAG-3 direct targeting is exploited by the use of a soluble dimeric recombinant LAG-3 (Eftilagimod alpha or IMP321), that stimulates DC through MHC class II molecules and induces sustained immune responses together with anti-PD-1, in patients with previously untreated unresectable or metastatic NSCLC (NCT03625323). Other approaches use bispecific antibodies targeting on one hand LAG-3 and on the other PD-1 (NCT04140500; NCT03219268), rather than single-agent compounds (NCT03250832; NCT03849469). More recently, the anti-LAG-3 antibody relatlimab (BMS-986016) has been assessed in the randomized, phase III trial RELATIVITY-047 in which 714 treatment-naïve patients affected by metastatic melanoma were randomized to receive nivolumab plus relatlimab or nivolumab plus placebo. Median PFS (the primary end-point) was significantly longer in the combination arm compared to the control arm (10.1 vs. 4.6 months; HR= 0.75; p= 0.0055); furthermore, the combination was well tolerated in terms of safety with no unexpected toxicities. Notably, RELATIVITY-047 is the first randomized study to demonstrate clinical benefit of dual LAG-3 and PD-1 inhibition in a solid tumor (141). Following these results, additional studies involving the dual blockade in other solid tumors, including NSCLC, are currently ongoing (NCT04623775) (**Table 2**).

TIM-3, apart from CTL, NK and Treg, is also expressed on DC and macrophages (in which its expression favors M2 polarization) (142). Monoclonal antibodies targeting TIM-3 either alone or in association with anti-PD-1 are under investigations in different clinical trials in solid tumors (NCT03652077; NCT02608268) (**Table 2**). Additionally, the use of bispecific antibodies capable to bind to both TIM-3 and PD-1 is being explored in ongoing trials specifically involving NSCLC patients (NCT03708328; NCT04931654). The safety and tolerability of combinations including anti-TIM-3 and anti-PD-1 with platinum-based doublet chemotherapy are currently being assessed in NCT03307785, and data collection is still on-going. Combination therapies simultaneously targeting TIM-3, PD-1 and LAG-3 immune checkpoint have also been evaluated for advanced cancers (NCT04641871). To date, only few clinical data are available for NSCLC. In a single-arm, phase II dose-expansion part of a phase I/II study, 33 patients (including 16 patients with melanoma and 17 with NSCLC), who were progressing after PD-1/PD-L1 blockade, received MBG453 (anti-TIM-3) and spartalizumab (anti-PD-1) until progression, death, or unacceptable toxicity. The combination resulted generally safe, but with limited activity in the setting of NSCLC and melanoma patients who had previously received ICI (143). Although definitive data are still immature, other early reports suggest that the combination of anti-TIM-3 (TSR-022) and anti-PD-1 (TSR-042) has shown activity in NSCLC patients progressing on previous anti-PD-1 therapy (142). Additionally, the anti-TIM-3 agent LY3321367 was employed alone (23 patients) or in combination with the anti-PD-1 antibody LY3300054 (18 patients) in a phase Ia/Ib trial (NCT03099109) (**Table 2**). Both combination and single-agent were well tolerated, and single-agent treatment with LY3321367 achieved > 20% tumor regression in two patients, one of which, affected by small cell lung cancer, was later confirmed as a partial response (144).

B7-H3, also known as CD276, is a transmembrane protein frequently expressed by cancer cells, and is considered an immune-checkpoint molecule exploited by cancer cells to escape immune system recognition. B7-H3 expression was hypothesized to be potentially involved in resistance to anti-PD-1/PD-L1 blockade in NSCLC (145). So far, 3 clinical trials assessed the possible use of an antibody to target B7-H3 in association with anti-PD-1 or anti-CTLA-4 in advanced, previously treated solid tumors (NCT03729596; NCT02475213; NCT02381314), while other studies are exploring the possibility to target B7-H3 by using Chimeric Antigen Receptor T Cells (CAR-T) (NCT03198052; NCT04842812). All these studies are currently ongoing.

T-cell immunoreceptor with Ig and ITIM domains (TIGIT) is expressed by activated CD8+ and CD4+ T cells, NK, Treg, and potently inhibits innate and adaptive immunity (146). While the mechanism of action of TIGIT has to be elucidated yet, the molecule is known to bind CD155, thus preventing its binding to the immune activator receptor CD226, down-regulating NK and T cells function. Furthermore, TIGIT is known to induce M2 macrophage differentiation (147). To date, the most promising anti-TIGIT agent in NSCLC is represented by tiragolumab. Recently, this agent has been evaluated in combination with atezolizumab in the CITYSCAPE trial. In this randomized, double-blind, phase II study, 135 previously untreated patients with advanced NSCLC positive for PD-L1 expression (≥1%) were randomized to receive tiragolumab plus atezolizumab or placebo plus atezolizumab as first-line treatment. In the intent-to-treat (ITT) population, objective response rate (ORR) was higher in the tiragolumab-atezolizumab arm compared to placebo-atezolizumab (37% vs. 21%). In sub-group analyses, the ORR advantage was confirmed in the subset of patients with PD-L1 expression ≥50% (ORR: 66% vs. 24%), while in the sub-group of patients with PD-L1 expression ranging from 1-49%, no advantage in terms of ORR was observed for the combination compared to placebo arm (16% vs. 18%). Similarly, a significant advantage in PFS was observed in the sub-group with PD-L1 ≥50% (median PFS not reached in the experimental arm compared to 4.11 months in the placebo arm; HR= 0.30), while no difference was observed in the sub-group with PD-L1 ranging from 1-49% (4.04 months vs. 3.58 months; HR= 0.89) (148).

With regards to other investigational agents, a currently ongoing phase II study aims to set safety and efficacy of zimberelimab (anti-PD-1) in combination with domvanalimab (anti-TIGIT) and etrumadenant (selective, A2A and A2B adenosine receptor, small-molecule antagonist) in previously treated 30 NSCLC patients (NCT04791839) (**Table 2**). This is an interesting approach to reduce inhibition of T and NK cells due to immune checkpoints and reduce adenosine mediated immunosuppression.

KIR expression in NSCLC was correlated to resistance to anti-PD-1 ICI (149). In a phase I-II clinical trial safety, tolerability, and efficacy of Epacadostat (IDO1 inhibitor), nivolumab (anti-PD-1), and lirilumab (anti-KIRD2) combination was evaluated on 11 patients with solid tumors (NCT03347123) (**Table 2**). Results are awaited with interest, though the number of patients included in the trial is small. Notably, increasing interest has raised towards the Natural-killer group 2 member A (NKG2A) receptor, which is typically expressed on NK cells and is characterized by inhibitory functions, although its mechanism of action is not yet fully disclosed (150). Recently, in the open-label, randomized, phase II COAST trial, 189 patients affected by inoperable, stage III NSCLC candidate for maintenance after chemo-radiation were randomized to receive either durvalumab (the current standard of care anti-PD-L1 agent) alone, durvalumab plus oleclumab (an anti-CD73), or durvalumab plus monalizumab (an anti-NKG2A). In the experimental arm including durvalumab plus monalizumab, ORR (the primary end-point) was superior than the standard arm including durvalumab alone (37.1% vs. 25.4%; Odds Ratio= 1.77). Similarly, durvalumab plus monalizumab achieved longer PFS compared to durvalumab alone at the interim analysis (15.1 vs. 6.3 months; HR= 0.65), thus suggesting a promising clinical role for the combination of PD-L1 and NKG2A inhibition (151).

### 5.2 Targeting Immune Suppression

Since the immune system is regulated by several immunosuppressive mechanisms, which represent interesting targets for novel agents designed to improve the activity of “classic” ICI. Such mechanisms and pathways are globally mediated by inflammatory regulators, metabolic regulators, as well as immunosuppressive cells within the TME, such as Treg and TAM (**Table 2** and **Figure 1**).

#### 5.2.1 Manipulation of Inflammatory Regulators

Cyclooxygenase (COX)-2 is frequently expressed by NSCLC and is required for prostaglandins synthesis, which are known to induce FoxP3+ Treg cells (152). Targeting COX-2 to inhibit Treg cells expansion and mediated immunosuppression has been exploited in several clinical trials using inhibitors in association with chemotherapy. Unfortunately, results did not meet the expectations. More specifically, in the GEmcitabine-COxib in NSCLC (GECO) study, the addition of oral rofecoxib to cisplatin-gemcitabine was associated with significantly increased rate of adverse events, including diarrhea, weight loss, constipation, fatigue and pain, as well as severe cardiac ischemia, without evidence of survival advantage (153). In the CALGB 30801 trial, 312 patients affected by unresectable NSCLC expressing COX-2 at immunohistochemistry assay were randomized to receive platinum-based chemotherapy with either celecoxib or placebo; the study was closed early due to futility as the addition of celecoxib failed to improve PFS over chemotherapy plus placebo (154).

While prospective data involving the use of ICI and COX inhibitors are limited, in a recent paper Wang et al. reported that the concomitant usage of COX inhibitors during ICI therapy for patients with NSCLC improved patients’ outcomes in terms of response (ORR at 6 months 73.7% vs 33.3%, p=0.036) and time to progression (HR 0.45; 95% CI 0.21 to 0.97; p=0.042), albeit these results were observed retrospectively in a cohort of 37 patients (155).

Targeting of TGF-β in association with ICI has been investigated using a bifunctional fusion protein (bintrafusp; M7824) consisting of the extracellular domain of TGF-β receptor II fused to an anti-PD-L1 in patients with NSCLC in a phase I trial. The expansion cohort of the trial included 80 NSCLC patients previously treated with platinum-based chemotherapy who were randomized at a one-to-one ratio to receive either bintrafusp alfa 500 mg or the recommended phase 2 dosage of 1200 mg every 2 weeks. The ORR was 17.5% and 25% in the 500 mg and 1200 mg dose, respectively; notably, ORR was higher in the sub-group of patients with PD-L1 expression ≥80% (ORR: 85.7%). The treatment was relatively well tolerated, with 69% of patients experiencing adverse events, including 23 out of 80 patients experiencing grade ≥3 adverse events (156). Other new studies are ongoing: in a phase II trial (NCT04396535) (**Table 2**) docetaxel is administered with or without bintrafusp alfa in treating patients after progressing on a combination of anti-PD-1/PD-L1 and chemotherapy; in a phase III trial (NCT03631706) the efficacy of bintrafusp alfa will be compared with pembrolizumab in patients with high PD-L1-tumor expression and no genetic alterations.

#### 5.2.2 Manipulation of Metabolic Mediators

Notably, some metabolic mediators, such as adenosine, arginine, and tryptophan (and its catabolic products) are involved in several immune-regulatory pathways, usually with immunosuppressive activity; hence, the pathways involving these molecules represent a promising target for immune-modulating treatments.

CD39/CD73 expressed on Treg and MDSC cells are considered another potential therapeutic target (36), indeed, multiple clinical trials designed to explore the activity of antibodies targeting either CD39 or CD73 in association with ICI alone or with chemotherapy are currently active and recruiting (NCT04306900, NCT03884556). Recently, results of the aforementioned COAST trial have been reported at the European Society of Medical Oncology (ESMO) Congress 2021; one of the treatment arms included in the trial was durvalumab plus oleclumab (an anti-CD73). This investigational combination was superior to durvalumab alone both in terms of ORR (38.3% vs. 25.4%; Odds Ratio= 1.83) and PFS (median not reached vs. 6.3 months; HR= 0.44) (151).

Arginase depletes arginine from tumor milieu and is produced by MDSC and neutrophils. Arginine is a fundamental amino acid which is required for optimal T cell functions (35); therefore, inhibition of arginase in association with ICI is apparently a potentially useful therapeutic approach for cancer immunotherapy. INCB001158 is a new inhibitory molecule of arginase, currently under investigation in a phase I clinical trial both as a single agent and in combination with “classic” ICI in patients with advanced/metastatic solid tumors [(157) NCT02903914] (**Table 2**). Results involving NSCLC have not been published yet, but the first data from patients with colorectal cancer indicate a good tolerability of INCB001158 in association with pembrolizumab and an increase in CD8+ T cells accumulation within the tumor (158, 159).

IDO1 and tryptophan 2,3-dioxygenase 2 (TDO2) catalyze the kynurenine metabolic pathway which leads, through tryptophan depletion in TME, to the generation of immune-tolerant DC and Treg, while the catabolic products kynurenines exert toxic activity on cytotoxic T cells (160, 161). Combination of epacadostat and pembrolizumab have largely disattended previous expectations in melanoma, and subsequently a phase II clinical trial investigating its potential activity in combination with pembrolizumab alone for treatment-naïve PD-L1 high (≥50%) NSCLC patients has been discontinued due to lack of advantage compared to pembrolizumab alone (NCT03322540). However, combinations of anti-PD-1 with other IDO-1 inhibitors (BMS-986205, NLG-919, navoximod/GDC-0919), dual IDO/TDO inhibitors (RG70099 and IOM-D) as well as indoximod are in clinical development (NCT03343613, NCT03322540, NCT02298153, NCT03562871) (**Table 2**).

#### 5.2.3 Manipulation of Immunoregulatory Cells

One possible approach for improving immune response to tumor relies in the modulation of immunoregulatory cells within the TME, with specific reference to immunosuppressive cells, which might be managed either directly (*e.g.* by depletion) or by reducing their proliferation (*e.g.* by use of inhibitors).

Since Treg are the immunosuppressive cells more frequently associated to resistance to ICI, the possibility of disrupting Treg function in association with ICI has been pursued. One possible approach is represented by the use of anti-CD25 antibody to deplete Treg in cancer. Currently, a single-arm phase Ib clinical trial exploiting the inhibition of Treg in association with pembrolizumab in different cancers, including NSCLC, is open for recruitment (NCT03621982). Patients enrolled in this study will receive ADCT-301/Camidanlumab tesirine, which is an anti-CD25 antibody–drug conjugate; the agent will be employed either alone or in combination with pembrolizumab. Preclinical studies demonstrated that this molecule would efficiently deplete Treg and cause immunogenic cell death and would concomitantly increase the number of activated tumor-infiltrating CD8+ T effector cells (162).

Recently, the results of a phase I trial involving the CD40 agonist APX005M (sotigalimab) and cabiralizumab, an inhibitor of colony stimulating factor-1 receptor (CSF1R), were published. Notably, CSF1R signaling is known to facilitate recruitment and activation of TAM and is associated with lower levels of cytotoxic lymphocytes, thus favoring an immunosuppressive environment (163); CD40 is a member of the TNF receptor super-family and is known to facilitate T cell activation and support a pro-inflammatory environment, including macrophage polarization towards M1 (164). In the trial, 26 patients with solid tumors, including 12 melanomas, 1 NSCLC, and 13 renal cell carcinomas, who had progressed on anti-PD-1/PD-L1 treatment, were treated in dose-escalating cohorts of APX005M with fixed doses of cabiralizumab, with or without nivolumab. The combination was generally tolerated and the observed results globally encourage further research involving combinations designed to polarize TME towards a pro-inflammatory pattern (164–166).

Another promising therapeutic target is represented by chemokine receptor type 4 (CCR4) known to stimulate the enrollment of Treg, thus promoting an immunosuppressive TME; hence, inhibiting CCR4 might result in Treg depletion and reversion towards immunogenic microenvironment. Mogamulizumab (anti-CCR4 antibody) has been evaluated in combination with anti-PD-1/PD-L1 or anti-CTLA-4 in two phase I trials. In the first trial, 96 patients with solid tumors received nivolumab plus escalating doses of mogamulizumab; the combination was generally safe, with mostly mild and moderate adverse events and no unexpected toxicities, and moderately active in terms of response, with 4 out of 15 patients with hepatocellular carcinoma achieving partial response. Among the 15 patients with NSCLC, 3 partial responses and 3 disease stabilizations were observed as best response (167). In the other phase I trial, 40 patients with solid tumors were included in dose-escalation cohorts of mogamulizumab concurrently with dose escalation of durvalumab or tremelimumab, and further 24 patients were included in dose-expansion cohorts. Although the combination treatment was generally well tolerated, the observed antitumor activity of mogamulizumab with either durvalumab or tremelimumab was modest across the different solid tumors involved (168).

### 5.3 Targeting Angiogenesis

Anti-angiogenic agents have been a mainstay among cancer therapies, with several compounds approved for multiple malignancies, either as “pure” anti-angiogenic agents, such as antibodies (bevacizumab, ramucirumab), or as multi-targeted agents active on angiogenesis as well as different molecular pathways (nintedanib, sunitinib, and others). The cornerstone of anti-angiogenic agents is currently represented by activity on VEGF and its receptors. Following the large use of angiogenesis-disrupting agents, great interest has developed toward the use of combinations of ICI and anti-angiogenic drugs. One notable difficulty associated with this approach lies in the necessity of equilibrium when formation of blood vessels is manipulated. Indeed, neo-angiogenesis promoted by tumor cells is typically chaotic and composed by disorganized and tortuous blood vessels characterized by excessive permeability, which results in increased interstitial fluid pression and ultimately reduced perfusion and oxygenation. Disrupting this process might result in transient normalization of blood circulation, thus facilitating the recruitment of lymphocytes. On the other hand, when anti-angiogenesis effects proceed, leucocytes have more difficulties in terms of accessibility to tumor mass, potentially resulting in less TILs (169). Notably, it has also been observed that high expression of VEGF results in increased proportion of immature DC, which promote immune tolerance, and Treg; furthermore, it has been suggested that VEGF might have a role in polarizing macrophages to M2 phenotype (169, 170). Recent updates on pre-clinical rationale and clinical experience with anti-VEGF agents and ICI have been comprehensively summarized by Ren et al. (48). The combination of the anti-VEGFR2 (ramucirumab) plus pembrolizumab in NSCLC was evaluated in a phase Ia/Ib trial. In an expansion cohort of the study, 11 out of 26 NSCLC patients (42.3%) experienced grade ≥3 treatment-related adverse events, the most frequent being hypertension (4 patients; 15.4%), which was consistent with the expected toxicity from ramucirumab; furthermore, 2 patients (7.7%) experienced myocardial infarction. Notably, ORR was 42.3% in the whole cohort, and patients with PD-L1 ≥50% achieved an ORR= 56.3%, compared to 22.2% achieved by the other patients. Similarly, median PFS was not reached for high PD-L1 expressors, while it was 4.9 months for patients with PD-L1 = 1-49% (171).

The combination of bevacizumab plus chemo-immunotherapy with atezolizumab, carboplatin, and paclitaxel was assessed in the large, randomized, phase III trial Impower150. In this study, which enrolled 1202 patients, the combination including bevacizumab achieved superior outcomes compared to the arm containing only bevacizumab, carboplatin, and paclitaxel, both in terms of PFS (8.3 vs. 6.8 months; HR= 0.62; p<0.001) and OS (19.2 vs. 14.7 months; HR= 0.78; p=0.02) (172). Notably, the trial included a small, although non-negligible sub-group of patients with activating mutations of *EGFR*, which are known to be associated with poor response to ICI. In this sub-population (124 patients), the combination of chemo-immunotherapy plus bevacizumab was associated with increased OS (median not reached at the time of analysis) over chemotherapy and bevacizumab alone (18.7 months), thus suggesting a potential effect of anti-angiogenesis plus ICI and chemotherapy in a population typically not suitable for treatment with ICI alone (173).

Finally a new and interesting approach targeting VEGF investigated the possible therapeutic efficacy of AK112, a PD-1/VEGF bispecific antibody, in patients with advanced NSCLC. The study is currently recruiting and its results are awaited (NCT04900363).

### 5.4 Targeting Cancer Cell Death

The possibility to target and inhibit Poly (ADP-ribose) polymerases (PARPs), thus triggering cell death in association with ICI to activate T cells represents an additional promising therapeutic approach; however, published data in NSCLC are still limited so far. In the phase II, JASPER trial, 38 patients affected by advanced NSCLC were divided in two cohorts (cohort 1: PD-L1 ≥50%; cohort 2: PD-L1=1-49%) and received first-line treatment with pembrolizumab plus niraparib. The primary end-point, ORR, was 56.3% in cohort 1 (9/16 evaluable patients) and 20.0% in cohort 2 (4/20 evaluable patients); with regards to survival outcomes in cohort 1 and cohort 2, median PFS was 8.4 months and 4.2 months, respectively, while OS was not reached and 7.7 months, respectively. Notably, 35.3% and 23.8% of patients in cohort 1 and cohort 2 experienced serious treatment-related adverse events. The authors concluded that the combination of pembrolizumab and niraparib is active in advanced NSCLC with high PD-L1 expression (174).

While published data involving PARP-inhibitors and ICI are still limited, several clinical trials are currently ongoing and might produce interesting results in the upcoming months. With regards to olaparib, the ongoing phase II ORION trial (NCT03775486), is evaluating the efficacy and safety of a maintenance with olaparib plus durvalumab combination compared to durvalumab alone in patients affected by stage IV NSCLC not progressing after a first-line of platinum-based chemotherapy plus durvalumab. Furthermore, two other phase III trials are evaluating the combination of pembrolizumab plus olaparib in NSCLC patients (NCT03976323, NCT03976362) (**Table 2**).

Finally, an ongoing phase I clinical trial aims to study the combination of niraparib (another PARP-inhibitor), TSR-022 (anti-TIM-3), bevacizumab, and platinum-based doublet chemotherapy with TSR-042 (anti-PD-1) in advanced or metastatic cancers, including NSCLC (NCT03307785) (**Table 2**). The mail goal of this study is to determine tolerability and safety of such combinations for subsequent phase II development.

## 6 Discussion

To date, ICI are the standard of care, either as monotherapy or in combination, for advanced non-oncogene-addicted NSCLC patients. However, a portion of patients do not benefit from these treatments and it is increasingly clear that reverting T or NK cytotoxic cell dysfunctional state with anti-PD-1/PD-L1 and/or anti-CTLA-4 may not be enough and needs to be improved. Indeed, increasing evidences sustain the role of new additional inhibitory immune checkpoint molecules, such as TIM-3, LAG-3, and TIGIT, in order to overcome the resistance to ICI (141, 144, 148). More importantly, the presence of an immune suppressive TME, mainly composed by Treg, MDSC and M2-TAM, in which cytotoxic cells reinvigorated by ICI act, is still a limitation for their anti-tumor activity, thus being acknowledged as another mechanism of resistance to ICI (32, 33, 37, 42, 43). Nonetheless, the identification of TILs with antigen specificity in the TME indicates that tumor recognition may occur and may lead to tumor growth control in the presence of an appropriate immune context (22). Further studies using multiplex histopathological, immunofluorescence and single-cell transcriptomics analyses are required to better define additional soluble mediators, cell to cell, and spatial relationships within the TME, that might collaborate to confer resistance to ICI. Moreover, an open question is how to select which patient will respond to treatment. Consequently, defining reliable biomarkers capable of predicting efficacy is a fundamental requirement. Currently, a number of TME-based scores both directly on tumor tissue visualization or indirectly through deductive techniques (*e.g.* gene expression profiles and radiomic feature extraction), have been tested as predictor of ICI efficacy in the lung cancer. Among these, PD-L1 expression by immunohistochemistry is still the only valid bio-marker widely used for the selection of suitable patients for anti-PD-1 treatment. Unfortunately, a number of issues are unresolved, such as the high intra-tumoral heterogeneity of PD-L1 which could prevent proper evaluation in small tumor biopsies, and pathologist interpretation is still a relevant factor (175). Gene signatures are now under development and show, for example, how an inflammatory state or the enrichment of the IFN pathway are predictors of a benefit from anti-PD-1/PD-L1 treatments. These predictive models have shown an optimal ability to retrospectively discriminate a benefit in disease response or progression, but prospective multi-institutional studies on larger patient cohorts are needed to ensure their reliability in a clinical setting.

To date multiple trials are currently ongoing with the aim of evaluating the use of novel agents in combination with ICI to overcome resistance (141–144, 146, 148). While these agents vary in terms of specific mechanism of action and some are explicitly designed to target additional immune checkpoints, other compounds are more specifically designed to interfere with TME (151, 153–156, 167, 171, 174). These approaches pursue the stimulation of a more pro-inflammatory microenvironment, usually by manipulating the proportion of immune cells populating the TME. More specifically their aim is the reduction of Treg and immature DC, while simultaneously favoring macrophage polarization toward an M1 differentiation rather than M2. It is important to stress that much of our current knowledge on resistance mechanisms and its biomarkers is derived from melanoma studies, and further studies, specific to lung cancer, are required.

Most clinical data are still limited so far, however, some interim results and safety data from phase I trials are already available and appear to be quite encouraging, especially when multi-modality approaches involving combinations of “classic” anti-PD-1/PD-L1 or CTLA-4 agents and novel immune-modulating drugs are employed. Data from ongoing clinical trials identify new interesting and promising drugs, such as tiragolumab (anti-TIGIT antibody) that in association with atezolizumab demonstrated higher ORR compared to placebo-atezolizumab (148). Other promising agents include monalizumab (anti-NKG2A antibody) and oleclumab (anti-CD73 antibody), both demonstrating to be superior to durvalumab alone, in terms of ORR and PFS (151). Similarly, other innovative immunotherapies, such as CAR-T or CAR-NK with selected tumor antigen specificity seem promising, and might represent a novel and effective approach to solid tumors (NCT04489862, NCT04153799, NCT02839954) (136, 137).

In the near future, we can expect that at least some of the currently investigated novel agents targeting additional immune checkpoints or components of the TME will proceed toward late phases of clinical research and eventually be approved. One potential issue will be represented by proper patient selection for receiving one among the different regimens that are available (single-agent PD-1 or PD-L1 inhibitor, chemotherapy plus PD-1/PD-L1 inhibitor, dual checkpoint blockade with PD-1 and CTLA-4 inhibitor plus chemotherapy), or among the regimens that might become available in the following months or years (such as PD-1 inhibitor plus TIGIT inhibitor, PD-1 inhibitor plus PARP inhibitor, among others). This is a most likely scenario for the next future, and we can also hypothesize that one strong focus of the upcoming research will be dedicated to the identification of predictive biomarkers of efficacy for the current and future regimens, eventually in addition or in replacement of PD-L1 expression. While the approach considering specific biomarkers and agents is intuitive *(e.g.* BRCA mutations and PARP inhibitors) and is easily accepted and adopted by pulmonary oncologists we have to consider that the addition of novel tissue-based biomarkers to the current panels of molecular alterations (which are expected to enlarge in their turn) might be severely limited by the amount of available adequate samples, especially since tissue will be consumed by routine molecular analyses. Furthermore, small biopsies might not be effectively representative of the complex interactions between the whole tumor and the immune system, and these interactions may change during time.

In conclusion, the exploitation of TME for the development of novel therapeutic strategies involving the components of TME, might represent the future of cancer immunotherapy. Moreover, the development of algorithms integrating clinical, histological, genetic, and radiomic features could help clinicians in patient management in defining specific personalized therapies comparable to what has been successfully done in oncogene-driven NSCLC.

## Author Contributions

CG, CD, and PP, introduction and clinical trials. PC and GuF, immunobiology. SS and SC, signatures. RG, GiF, and MC, tumor microenvironment. CG, SC, RG, and MC, discussion. All authors contributed to the article and approved the submitted version.

## Funding

The authors wish to thank the Italian Ministry of Health (5x1000 funds, Ricerca Corrente, CO-2016-02361470), Compagnia di San Paolo (2017–0529), AIRC 5x1000 2018 Id:21073, and Bristol-Myers-Squibb, which provided grants in support to our research in cancer immunotherapy. The funder was not involved in the study design, collection, analysis, interpretation of data, the writing of this article or the decision to submit it for publication.

## Conflict of Interest

CG declares honoraria from Astra Zeneca, Bristol-Myers-Squibb, Boehringer-Ingelheim, Merck-Sharp-Dohme, Roche, Takeda. CD declares honoraria from Astra Zeneca, Bristol-Myers-Squibb, Merck-Sharp-Dohme, Roche.

The remaining authors declare that the research was conducted in the absence of any commercial or financial relationships that could be construed as a potential conflict of interest.

## Publisher’s Note

All claims expressed in this article are solely those of the authors and do not necessarily represent those of their affiliated organizations, or those of the publisher, the editors and the reviewers. Any product that may be evaluated in this article, or claim that may be made by its manufacturer, is not guaranteed or endorsed by the publisher.
